# Improved pre-operative diagnostic accuracy for low-grade prosthetic joint infections using second-generation multiplex Polymerase chain reaction on joint fluid aspirate

**DOI:** 10.1007/s00264-020-04552-7

**Published:** 2020-04-15

**Authors:** Christian Suren, Susanne Feihl, Sabrina Cabric, Ingo J. Banke, Bernhard Haller, Andrej Trampuz, Rüdiger von Eisenhart-Rothe, Peter M. Prodinger

**Affiliations:** 1 Klinik und Poliklinik für Orthopädie und Sportorthopädie, Klinikum rechts der Isar der Technischen Universität München, Ismaninger Str. 22, 81675 Munich, Germany; 2 Institut für Medizinische Mikrobiologie, Immunologie und Hygiene, Technische Universität München, Trogerstr. 30, 81675 Munich, Germany; 3 Centrum für Muskuloskeletale Chirurgie (CMSC), Charité-Universitätsmedizin Berlin, Charitéplatz 1, 10117 Berlin, Germany; 4 Institut für medizinische Informatik, Statistik und Epidemiologie, Technische Universität München, Grillparzerstr. 18, 81675 Munich, Germany

**Keywords:** PJI, Polymerase chain reaction, diagnostic, Diagnosis, Pre-operative, Arthrocentesis, Synovial fluid, Causative organism, Agent, Detection, DNA, Bacteria, Bacterial

## Abstract

**Background:**

A major obstacle for the treatment of prosthetic joint infection (PJI) is the identification of the underlying causative organism. While the diagnostic criteria ruling PJI in or out have become ever more accurate, the detection of the causative pathogen(s) still relies mostly on conventional and time-consuming microbial culture. The aim of this study was to evaluate the diagnostic potential of a second-generation multiplex PCR assay (*Unyvero* ITI G2, Curetis AG, Holzgerlingen, Germany) used on synovial fluid specimens. Our hypothesis was that the method would yield a higher diagnostic accuracy in the pre-operative workup than synovial fluid culture. Thus, a more precise classification of septic and aseptic prosthesis failure could be achieved before revision surgery.

**Methods:**

Prospectively collected frozen joint fluid specimens from 26 patients undergoing arthroplasty revision surgery of the hip or knee were tested as per the manufacturer’s protocol. Sensitivities, specificities, positive and negative predictive values as well as positive and negative likelihood ratios with corresponding confidence intervals were estimated using the statistical software *R*. A combination of the serum C-reactive protein (CRP) level, leukocyte count, erythrocyte sedimentation rate, joint fluid culture, tissue biopsy culture, and tissue biopsy histology served as the gold standard.

**Results:**

Of the 26 patients included in the study, 15 were infected and 11 were aseptic. Conventional joint fluid culture showed a sensitivity of 0.67 and a specificity of 0.91. Joint fluid multiplex PCR yielded a sensitivity of 0.8 and a specificity of 1.0.

**Conclusions:**

Using the second-generation Unyvero ITI cartridge on joint fluid aspirate for the detection of prosthetic joint infection, we were able to achieve a higher diagnostic accuracy than with conventional culture. We conclude that to improve pathogen detection before revision surgery, this method represents a valuable and practicable tool.

## Introduction

Prosthetic joint infection (PJI) is a serious complication of arthroplasty procedures with devastating consequences for patients and healthcare systems. The diagnosis of PJI is still challenging. As there is no single diagnostic parameter available, the diagnosis is made under the consideration of several pre- and intra-operative criteria [1–5]. However, the identification of the underlying pathogen still relies on standard cultures that are prone to false-negative results in the instance of fastidious organisms or false-positive results when the samples are contaminated [6–8]. Additionally, most sets of diagnostic criteria rely on the assessment of information gained intra-operatively, such as tissue biopsies, the sonication of explanted components, and histological workup of the synovia-like interface membrane (SLIM) [2, 9–14]. Thus, the diagnostic process that is set into motion pre-operatively might lack adequate information for a conclusive diagnosis before surgery [10]. As a consequence, infections can be missed, resulting in inadequate treatment, eventually leading to persistent infection with additional patient morbidity and the necessity of further surgical interventions [15]. On the other hand, infections can be falsely assumed, leading to overtreatment and surgery-related morbidity [16–18]. Therefore, more accurate diagnostic methods are needed not only to protect patients from harm but also to plan effective treatment strategies. Recently, molecular diagnostic methods such as polymerase chain reaction (PCR) have been used in this context [19–21]. With this method, the time required to identify the bacterium causing the infection can be reduced considerably. Furthermore, previous antibiotic treatment should have little or no impact on the identification process, as PCR does not necessarily require viable bacteria [22]. However, conventional broad-range PCR (16S ribosomal DNA PCR) is limited due to false-positive results from contamination and difficulties in the detection of mixed infections [23–26]. Multiplex PCR aims to balance this disadvantage by enabling the simultaneous detection of a whole panel of potentially causative organisms [22, 25, 27].

Again, the value of this diagnostic method depends in part on where in the diagnostic cascade it is put to use. While the combination of all conventional pre-operative and intra-operative diagnostic measures yields a sufficient diagnostic accuracy and the chance of the identification of the underlying organism, the identification of the bacterium in conventional cultures of synovial fluid collected pre-operatively often fails [3, 26, 28, 29]. This pre-operative diagnostic gap is reflected in the recently published diagnostic algorithm based on the 2018 MSIS criteria by Parvizi et al., in which the fulfilment of only some of the minor criteria leading to a score of 2–5 points is considered inconclusive, requiring the collection of tissue biopsies and histological analysis during surgery to completely rule out infection [10].

*Unyvero* (Curetis AG, Holzgerlingen, Germany), a commercially available automated multiplex PCR assay, has been evaluated with favourable results on synovial fluid, tissue and sonication fluid specimens in prosthetic joint infections [25, 30–36]. The new ITI (*Implant and Tissue Infection)* G2 cartridge generation introduced in 2016 offers an expanded panel of microorganisms and an enhanced sensitivity to detect fastidious organisms such as *Cutibacterium acnes* (formerly *Propionibacterium acnes*) (Tables 1 and 2). Our hypothesis was that the expanded panel of organisms would yield a higher diagnostic accuracy in the pre-operative workup. Thus, a more precise classification of septic and aseptic prosthesis failure could be achieved before revision surgery.

**Table 1:** Microorganisms of the Unyvero ITI G2 panel. The PCR product of the “Universal Bacteria” primer is detected by several bacterial probes

**Group**	**Pathogen**		**Group**	**Pathogen**		**Group**	**Pathogen**	
**Universal bacteria**			**Corynebacteriaceae**	**Corynebacterium spp.:**		**Non-fermenting bacteria**		
					*C. jeikeium*		***Pseudomonas aeruginosa***	
**Gram-positive bacteria**					*C. belfanti*		***Acinetobacter baumannii complex:***	
	***Staphylococcus aureus***				*C. amycolatum*			*A. baumannii*
					*C. striatum*			*A. oleivorans*
	**Coagulase-negative staphylococci:**				*C. aurimucosum*			*A. calcoaceticus*
		*S. saprophyticus*						*A. pittii*
		*S. hominis*	**Enterobacteriaceae**					
		*S. epidermidis*		***Escherichia coli***		**Anaerobes**		
		*S. warneri*		***Enterobacter cloacae*** **complex:**			***Cutibacterium acnes***	
		*S. haemolyticus*			*E. cloacae*		***Finegoldia magna***	
		*S. capitis*			*E. asburiae*		***Bacteroides fragilis*** **group:**	
		*S. lugdunensis*			*E. hormaechei*			*B. fragilis*
	**Streptococcus spp.:**			***Enterobacter aerogenes***				*B. thetaiotaomicron*
		*Streptococcus pneumoniae*		**Proteus spp.:**				*B. ovatus*
		*Streptococcus mitis*			*P. vulgaris*			*B. uniformis*
		*Streptococcus pyogenes*			*P. mirabilis*			
		*Streptococcus agalactiae*			*P. penneri*	**Fungi**	**Candida spp.:**	
		*Streptococcus sanguinis*			*P. hauseri*			*Candida albicans*
		*Streptococcus pyogenes / dysgalactiae*		***Klebsiella pneumoniae***				*Candida tropicalis*
		*Streptococcus dysgalactiae subsp. equisimilis*			*Klebsiella pneumoniae Cluster kp I + II*			*Candida orthopsilosis*
		*Streptococcus dysgalactiae subsp. dysgalactiae*			*Klebsiella pneumoniae subsp. ozaenae*			*Candida parapsilosis*
		*Streptococcus gordonii*			*Klebsiella oxytoca*			*Candida dubliniensis*
		*Streptococcus pneumoniae*			*Klebsiella variicola*			*Candida viswanathii*
		*Streptococcus agalactiae*			*Citrobacter freundii / koseri*			*Candida metapsilosis*
		*Granulicatella adiacens*						*Candida labiduridarum*
		*Abiotrophia defectiva*						
	**Enterococcus spp.*:***							
		*E. faecalis*						
		*E. faecium*						
		*E. gallinarum*						
		*E. casseliflavus*						
		*E. avium*						
		*E. hirae*						
		*E. durans*						
		*E. raffinosus*

**Table 2:** Resistance gene markers of the *Unyvero* ITI G2 panel

**Marker**	**Resistance**
**ermA**	Macrolides/lincosamides
**ermC**	Macrolides/lincosamides
**mecA**	Oxacillin/methicillin
**mecC (LGA251)**	Oxacillin/methicillin
**vanA**	Glycopeptides
**vanB**	Glycopeptides
**aac(6′)/aph(2″)**	Aminoglycosides
**aacA4**	Aminoglycosides
**ctx-M**	3rd-generation cephalosporin, class A
**imp**	Carbapenems, class B
**kpc**	Carbapenems, class B
**ndm**	Carbapenems, class B
**oxa-23**	Carbapenems, class D
**oxa-24/40**	Carbapenems, class D
**oxa-48**	Carbapenems, class D
**oxa-58**	Carbapenems, class D
**vim**	Carbapenems, class B

Therefore, the aim of this study was to evaluate the diagnostic accuracy of this second-generation assay on synovial fluid specimens collected pre-operatively.

## Patients and methods

The study protocol was approved by the institutional review board under reference no. 2544/09. Informed consent was obtained from every patient prior to screening. We used synovial fluid samples from a prospective cohort of patients who underwent revision surgery for total hip or knee arthroplasty at our institution in 2009 [37]. As per standard institutional procedure prior to arthroplasty revision, leukocyte count, C-reactive protein (CRP), erythrocyte sedimentation rate (ESR), joint aspirate culture, and joint aspirate leukocyte count and differential were obtained from all patients. During surgery, tissue biopsies for culture and histology were obtained. After the collection of the specimens, one specimen of joint aspirate and one tissue biopsy per patient were frozen and stored at − 80 °C for further examination. Automated multiplex PCR analysis was performed as per the manufacturer’s protocol after the specimens of joint fluid aspirate were thawed. The joint fluid sample was subjected to mechanical, thermal, chemical, and enzymatic lysis in a test tube for 30 minutes and then transferred into an “implant and tissue infection” (ITI) cartridge. The master mix, including deoxyribonucleic acid (DNA)-polymerase, primers, nucleotides and PCR-buffer, was added, and the cartridge was inserted into the *Unyvero Analyser* for the actual PCR. A result was reported as positive if an analyte reached the threshold of 10^4^ DNA fragments/pathogens/ml.

The patients enrolled were classified as either infected or aseptic after the histological and microbiological analysis of intraoperative tissue biopsies according to the standard then in use at our institution, which was implemented on the basis of the diagnostic criteria published by Zimmerli et al. [11]. Accordingly, a patient was classified as infected if there was a sinus tract present, if there was purulence around the joint or if a virulent organism was isolated in synovial fluid or tissue culture. Furthermore, a combination of any of the following findings led to the diagnosis of an infection: elevated serum CRP, blood leukocyte count or elevated ESR with the identification of a non-virulent organism in a joint fluid or tissue specimen, with positive histology as defined by Morawietz and Krenn [12], or the identification of a non-virulent organism with positive histology. This classification was used as the gold standard for the analysis of the diagnostic accuracy of the multiplex PCR.

For microbiological analysis, synovial fluid was cultivated on aerobic and anaerobic plates (Columbia sheep blood agar, Columbia chocolate agar, McConkey agar, Schädler anaerobic agar and Schädler KV anaerobic agar) and in liquid media (thioglycolate and glucose broth) at 37 °C in aerobic and anaerobic atmospheres. Media were checked for bacterial growth after 24 hours, 48 hours and ten days. Biochemical identification was performed with Vitek2 (bioMerieux, Nürtingen, Germany).

### Statistical analysis

The patients’ pre- and post-operative classification according to the diagnostic gold standard and the pre-operative diagnosis using multiplex PCR were recorded. Furthermore, the identified organisms and their respective resistance genes were collected and entered in an Excel table (Microsoft Corporation, Richmond, USA).

Absolute frequencies are given for categorical data, and the mean and minimum and maximum values are presented for age. Sensitivities, specificities, positive and negative predictive values as well as positive and negative likelihood ratios were estimated and presented. For sensitivity, specificity and predictive values, corresponding exact 95% confidence intervals were estimated based on the binomial distribution (Clopper-Pearson intervals [38]). For likelihood ratios, confidence intervals were estimated as described in [39]. All analyses were performed using the statistical software *R* (version 3.6.0) and its library *epiR* [40, 41].

## Results

Twenty-six patients (8 males, 18 females, 13 total hip arthroplasties, 13 total knee arthroplasties) with a mean age of 72.3 years (52.1–98.2 years) were included. PJI was diagnosed in 15 patients, and 11 patients were aseptic. The aseptic cases included four instances of aseptic loosening of total hip arthroplasties, four instances of aseptic loosening of total knee arthroplasties, two instances of malalignment in total knee arthroplasties, and one case of patella baja and arthrofibrosis in a total knee arthroplasty.

Of the 15 cases of infection, five had been misdiagnosed as aseptic preoperatively. In each case, the diagnosis was changed due to the additional intra-operative findings.

Joint fluid aspirates showed growth on conventional culture media in ten out of 15 PJI cases and in one of the 11 aseptic cases, yielding a sensitivity of 0.67 (0.23, 0.63) and a specificity of 0.91 (0.59, 1.00). In three of the five false-negative PJI cases, the organism that was later identified in intra-operative tissue biopsy culture could be identified via joint fluid multiplex PCR. There was one false-positive classification due to the growth of *Staphylococcus aureus* in the joint aspirate culture before surgery. This finding was not confirmed in later tissue biopsy cultures or histology.

Multiplex PCR identified pathogens in 12 out of 15 PJI cases and in none of the aseptic cases. This resulted in a sensitivity of 0.8 (0.52, 0.96) and a specificity of 1.0 (0.69, 1.0). Two of the PJI cases in which multiplex PCR failed to identify any organism also did not show any growth in conventional synovial fluid culture. The remaining false-negative PJI case showed the growth of group B streptococci in conventional culture of the synovial fluid as well as intra-operative tissue biopsies. For an overview of the positive and negative predictive values and likelihood ratios of joint fluid culture and joint fluid PCR, respectively, see Table 3.

**Table 3:** Overview of patient data, affected joint, preoperative and postoperative classification, diagnosis, and identified bacteria

**Patient no.**	**Age (years)**	**Sex**	**Hip/knee**	**Pre-operative classification**	**Diagnosis**	**Preoperative PCR**	**SF culture**	**SF PCR**	**Biopsy culture**
**1**	**80.1**	**f**	**Hip**	PJI	PJI	PJI	*S. epidermidis*	*CNS*	*S. epidermidis, S. hominis*
**2**	**54.1**	**f**	**Hip**	Aseptic loosening	PJI	PJI	*–*	*CNS*	*CNS*
**3**	**52.1**	**m**	**Knee**	PJI	PJI	PJI	*CNS*	*CNS*	*CNS*
**4**	**98.2**	**f**	**Knee**	PJI	PJI	PJI	*S. epidermidis*	*CNS*	*S. epidermidis*
**5**	**78.4**	**f**	**Knee**	Aseptic loosening	PJI	Aseptic	*–*	–	*CNS*
**6**	**66.7**	**m**	**Hip**	PJI	PJI	PJI	*2 strains CNS*	*CNS, Cutibacterium acnes*	*2 strains S. epidermidis*
**7**	**60.9**	**m**	**Hip**	Heterotopic ossification	PJI	PJI	*–*	*CNS*	*S. epidermidis*
**8**	**84.9**	**m**	**Hip**	PJI	PJI	PJI	*Listeria monocytogenes*	*CNS*	*Listeria monocytogenes*
**9**	**66**	**f**	**Knee**	PJI	PJI	PJI	*S. aureus*	*S. aureus*	*S. aureus*
**10**	**82.2**	**f**	**Hip**	PJI	PJI	PJI	*S. aureus*	*S. aureus*	*S. aureus*
**11**	**52.5**	**f**	**Knee**	Aseptic loosening	PJI	Aseptic	*–*	*–*	*CNS*
**12**	**75.6**	**f**	**Hip**	Aseptic loosening	PJI	PJI	*–*	*CNS*	*CNS*
**13**	**78.2**	**f**	**Knee**	PJI	PJI	PJI	*Streptococcus salivarius*	*Streptococcus spp.*	*Streptococcus salivarius*
**14**	**60.8**	**m**	**Hip**	PJI	PJI	PJI	*Group B Streptococci*	*Streptococcus agalactiae*	*–*
**15**	**83.3**	**f**	**Hip**	PJI	PJI	Aseptic	*Group B Streptococci*	*–*	*Group B Streptococci*
**16**	**81.7**	**f**	**Hip**	Aseptic loosening	Aseptic loosening	Aseptic	*–*	*–*	–
**17**	**73.5**	**f**	**Knee**	Malalignment	Malalignment	Aseptic	*–*	*–*	–
**18**	**77**	**f**	**Knee**	Aseptic loosening	Aseptic loosening	Aseptic	*–*	*–*	–
**19**	**71**	**m**	**Knee**	Aseptic loosening	Aseptic loosening	Aseptic	*–*	*–*	–
**20**	**77.4**	**f**	**Knee**	Malalignment	Malalignment	Aseptic	*–*	*–*	–
**21**	**83.2**	**f**	**Knee**	Aseptic loosening	Aseptic loosening	Aseptic	*–*	*–*	–
**22**	**79.2**	**f**	**Hip**	Aseptic loosening	Aseptic loosening	Aseptic	*–*	*–*	–
**23**	**64.1**	**m**	**Hip**	Aseptic loosening	Aseptic loosening	Aseptic	*–*	*–*	–
**24**	**59**	**f**	**Knee**	Aseptic loosening	Aseptic loosening	Aseptic	*–*	*–*	–
**25**	**67.8**	**m**	**Knee**	Arthrofibrosis	Arthrofibrosis	Aseptic	*–*	*–*	–
**26**	**74**	**f**	**Hip**	PJI	Aseptic loosening	Aseptic	*S. aureus*	*–*	–

*SF* synovial fluid

Multiplex PCR analysis identified resistance genes in five of the 12 detected pathogens. The *mecA* gene mediating methicillin resistance was found three times in coagulase-negative staphylococci. The *ermC* and *ermA* genes were found in coagulase-negative staphylococci and *S. aureus*, respectively. Both genes are resistance markers for macrolides and lincosamides. Phenotypical susceptibility testing confirmed all genotypically detected antibiotic resistances. Apart from the ones detected by PCR, there were no clinically relevant phenotypical resistances.

There were some discrepancies between the positive culture and PCR results. In one case, the growth of *Listeria monocytogenes* in fluid culture was found, while PCR was positive for coagulase-negative staphylococci (CNS). In the subsequent tissue culture, the growth of *Listeria monocytogenes* was confirmed, but not of CNS. In another case, fluid culture exhibited the growth of two strains of CNS, both later confirmed in tissue biopsies. *Unyvero* PCR, however, was positive for CNS and *C. acnes*. For an overview of the results, see Table 4.

**Table 4:** Overview of the diagnostic parameters (with 95% confidence intervals) of joint fluid culture and multiplex PCR

**Test**	**True positive**	**False positive**	**True negative**	**False negative**	**Sensitivity**	**Specificity**	**PPV**	**NPV**	**LR+**	**LR-**
**Joint fluid culture**	10	1	10	5	**0.67** (0.23, 0.63)	**0.91** (0.59, 1.00)	**0.91** (0.59, 1.00)	**0.67** (0.38, 0.88)	**7.33** (1.09, 49.16)	**0.37** (0.17, 0.77)
**Joint fluid multiplex PCR**	12	0	11	3	**0.8** (0.52, 0.96)	**1.0** (0.69, 1.0)	**1.0** (0.74, 1.0)	**0.77** (0.46, 0.95)	**n/a**	**0.2** (0.07, 0.55)

*PPV* positive predictive value; *NPV* negative predictive value; *LR+* positive likelihood ratio; *LR-* negative likelihood ratio

## Discussion

In our study, multiplex PCR of joint fluid aspirate specimens showed superior diagnostic accuracy to that of conventional culture, even when used on previously frozen and stored fluid specimens. If the method had been used at the time of the sample collection, three patients would have been correctly diagnosed with PJI based on pre-operative PCR results in joint fluid. Instead, the pre-operative diagnosis had to be changed after the results from intra-operative tissue cultures became known. Conversely, there was one false-negative result using the method on joint fluid.

The sensitivity and specificity of joint fluid aspirate culture in this study are comparable to those of reliable reports in the available literature (sensitivity 0.72–0.8 and specificity 0.93–0.95) [26, 42–44]. As joint fluid aspirate culture is currently the only widely applied means to pre-operatively identify the infectious agent causing PJI, the relatively low sensitivities in these reports underline our argument for additional or improved methods of detection.

Failure to detect the organism causing infection leads to the constellation of culture-negative PJI, which is challenging to treat [45]. The rate of culture-negative infections varies in the available literature, but it is considerable, ranging as high as 42% [46]. The ramifications of culture-negative PJI for the treatment strategy and the outcome are equivocal in most reports, with some authors even postulating a higher success rate when the causative microbe is unknown [47–49]. In fact, culture-negative PJI caused by fastidious organisms such as *S. epidermidis* and *C. acnes* might be successfully treated using the right empirical antibiotic regimen. However, when culture-negative PJI is caused by fungi, mycobacteria or other unusual agents, empirical therapy is likely to fail. This likelihood is reflected in a more recent study that found an impaired treatment success rate when the agent was unknown [50]. The authors concluded that the pre-operative identification of the underlying organism was paramount to successful treatment and should be pursued unconditionally.

In our opinion, the weakness of the currently accepted diagnostic standard lies in the low sensitivity of synovial fluid culture. As a result, both culture-negative PJI and falsely diagnosed “aseptic” loosening limit our treatment outcomes. The results of our study suggest that the diagnostic accuracy of pathogen detection in joint fluid can be ameliorated using multiplex PCR.

*Unyvero* multiplex PCR has been used on synovial fluid specimens before. Morgenstern et al. reported a slightly inferior sensitivity and specificity of multiplex PCR used in synovial fluid samples from 142 patients when compared to our findings [25]. However, they were using the first-generation *Unyvero* ITI cartridge. Our use of the second-generation cartridge might explain the results reported here. Lausmann et al. published results of the same first-generation test used on synovial aspirates from 60 patients, including patients with acute PJI [30]. Their reported sensitivity and specificity of 0.79 and 1.0, respectively, are comparable to our results. However, the inclusion of acute PJI cases is likely to have increased the diagnostic accuracy, as the much higher bacterial burden in acute PJI cases is more likely to be detected. In general, acute PJI is fairly easy to diagnose, and the identification of the causative agent by joint aspirate culture is reliable in those cases. Therefore, we focused on low-grade PJI, where the correct diagnosis and microbe identification often prove to be difficult, especially with the limited material obtainable pre-operatively via joint aspiration.

Conventional and multiplex PCR have been used on sonication fluid to improve the intra-operative capabilities of microbe identification. In a recent meta-analysis, neither method was shown to be superior to sonication fluid culture [27, 51]. In our view, this underscores the fact that the combination of all pre-operative and intra-operative criteria yields a high diagnostic value, and the well-established diagnostic algorithms barely need improvement [10, 26]. However, the pre-operative workup for the exclusion of low-grade infections based on serum inflammation markers and joint aspiration could profit from the implementation of molecular methods for microbe detection.

There were two discrepancies between the results of the PCR and those of conventional culture worth discussing. First, both conventional cultures of joint fluid and tissue biopsies exhibited infection with *Listeria monocytogenes* in one case. While the PCR failed to detect the same organism, it was positive for CNS. Interestingly, it is one of only two instances with a low signal intensity in the PCR. Furthermore, *Listeria monocytogenes* is not included in the *Unyvero* ITI panel of organisms; therefore, it could not have been correctly detected by it. Whether the positive result showing CNS was caused by sample contamination or the presence of coagulase-negative staphylococci undetected by conventional means is unclear. In the second discrepancy seen, conventional cultures of joint fluid and tissue biopsies detected two different strains of *S. epidermidis* in one patient. While *Unyvero* is per se unable to discern different strains of the same organism, it did show CNS and, in addition, *Cutibacterium acnes*. Again, whether this constitutes a relevant finding, a contamination, or a false-positive result remains unclear. Accordingly, we considered the detection of CNS a true positive result. Due to the fastidiousness of *C. acnes*, it is a possible scenario that the conventional culture failed to detect its presence. In that case, the PCR result would be a true positive.

We reported one patient pre-operatively classified as infected due to the growth of *S. aureus* in the joint aspirate culture who was later deemed to be aseptic. By default, any detection of a highly virulent agent such as *S. aureus* is considered to prove the presence of an infection. The patient was treated with a two-stage prosthesis exchange. However, there were no positive intra-operative biopsies, and the histological results were negative. Therefore, despite convention, we considered the patient to have been aseptic in retrospect.

There are some limitations of this study. First, while patient recruitment and pre-operative and intra-operative conventional diagnostics were performed prospectively, multiplex PCR analysis was performed on stored specimens retrospectively. While this restricts data quality, it might also have resulted in reduced specimen quality and hence poorer performance of the PCR. Specimen freezing and thawing leads to more viscous fluid samples, which need to be diluted in some instances [52]. Where the bacterial load is low, this dilution might lower the DNA count below the detection threshold and cause false-negative results. Apart from that, tests performed by the manufacturer showed no effects on pathogen detection after 1 year of storage at − 70 °C of both native and artificial spiked samples. In our collective, only one viscous sample had to be diluted, and it still yielded a positive result. As there were no false-negative readings in this study, we assume that the freezing and thawing of samples had no sizable detrimental effects.

Second, the diagnostic gold standard used in our institution in 2009, even though similar to the currently established standards, is dated. Applying one of today’s established diagnostic algorithms might change the results of our evaluation. However, there are currently several diagnostic algorithms for prosthetic joint infections existing in parallel. All of them have been and are still subject to changes and amendments; therefore, it remains problematic to define a gold standard when evaluating a new testing method. Even within the framework of a defined standard, “new” diagnostic tools, such as sonication, alpha-defensin or any DNA-based assays, are difficult to judge. As the diagnosis is established under the consideration of various factors, they will serve as a more or less meaningful amendment to what is already known.

Being well aware of the gold standard problem, we would like to emphasize that there is a requirement to improve our ability not just to diagnose PJI but also to identify the agent causing it *before* a treatment is initiated. Based on our findings, multiplex PCR could represent a useful tool for just that. Using multiplex PCR on aspirated joint fluid might prove very helpful in addition to joint aspirate culture to increase the chance of pathogen identification before surgery.

## Abbreviations

CNS: Coagulase-negative staphylococci
CRP: C-reactive protein
DNA: Deoxyribonucleic acid
EBJIS: European Bone and Joint Infection Society
ESR: Erythrocyte sedimentation rate
ITI: Implant and tissue infection (cartridge)
LR: Likelihood ratio
MSIS: Musculoskeletal Infection Society
NPV: Negative predictive value
PCR: Polymerase chain reaction
PJI: Prosthetic joint infection
PPV: Positive predictive value
SLIM: Synovia-like interface membrane
